# Multidimensional contributors to disease burden in axial spondyloarthritis: role of central sensitization, catastrophizing and sleep disturbance

**DOI:** 10.1186/s42358-025-00512-0

**Published:** 2026-01-12

**Authors:** Jeniffer Mirelli dos Santos Lopes, Aline Ranzolin, Nara Gualberto Cavalcanti, Angela Luzia Branco Pinto Duarte, Claudia Diniz Lopes Marques

**Affiliations:** 1 Programa de Pós-Graduação em Saúde Translacional, Universidade Federal de Pernambuco, Recife, Pernambuco Brazil; 2 Department of Rheumatology, Hospital das Clínicas da Universidade Federal de Pernambuco/Ebserh, Recife, Pernambuco Brazil

**Keywords:** Axial spondyloarthritis, Central sensitization, Pain catastrophizing, Disease activity

## Abstract

**Introduction:**

Axial spondyloarthritis (axSpA) imposes a multidimensional burden that is not fully explained by inflammation. Central sensitization (CS), pain catastrophizing (PC), and sleep disturbance may amplify symptoms and worsen outcomes. This study aimed to assess the prevalence and impact of CS, PC, and sleep disturbances in axSpA patients and their associations with disease activity, function, and quality of life compared with controls.

**Methods:**

This cross-sectional study included adults with axSpA (ASAS 2009) and healthy controls recruited from a tertiary clinic (April 2024–April 2025). The assessments included demographics; CSI, PCS, JSS, fibromyalgia (ACR-2016), fibromyalgianess (WPI + SSS); and axSpA outcomes (BASDAI, ASDAS, BASFI, BASMI, ASQoL, CRP, and MASES). Statistical analyses included group comparisons, correlations, and multivariable regressions.

**Results:**

We enrolled 100 axSpA patients and 50 controls. The median scores were greater in the axSpA patients for the CSI (42 vs. 28), PCS (32 vs. 12.5), and JSS (12 vs. 6) (all *p* < 0.001). The prevalence was greater for CS (59% vs. 20%), PC (53% vs. 18%), and fibromyalgia (43% vs. 18%). The WPI was strongly correlated with the SSS (*r* = 0.92). In the axSpA patients, the CSI was correlated with the BASDAI (*r* = 0.58), ASDAS (*r* = 0.43), BASFI (*r* = 0.45), and ASQoL (*r* = 0.68) (all *p* ≤ 0.001). The PCS and JSS are also correlated with disease activity, disease function, and ASQoL. Independent predictors were CSI—female sex, higher SSS, and worse ASQoL; PCS—ASQoL; JSS—higher SSS and arthritis, with lower scores in patients on TNF inhibitors or pain-modulating therapy.

**Conclusion:**

CS, PC, sleep disturbance, and FM/FMness are highly prevalent in axSpA patients and are independently associated with worse outcomes. Incorporating nociplastic and psychosocial dimensions into assessment and care is crucial to reduce disease burden.

## Introduction

Axial spondyloarthritis (axSpA) is a chronic immune-mediated disease that primarily affects the sacroiliac joints and spine and is characterized by pain, stiffness, and progressive functional impairment [1]. Although inflammation is central to its pathogenesis, many patients continue to report pain, fatigue, and sleep disturbances despite targeted therapy, suggesting that inflammatory control alone does not fully account for disease burden [2, 3].

Emerging evidence indicates that noninflammatory mechanisms play a critical role in symptom severity. Central sensitization (CS), defined as amplification of nociceptive signaling within the central nervous system, is associated with disproportionate pain and reduced treatment satisfaction. Patients with axSpA have worse sleep quality and higher CS scores than healthy controls do, and CS has been linked to increased disease activity, functional impairment, and poorer quality of life [4, 5].

Pain catastrophizing (PC), a maladaptive cognitive-emotional response involving rumination, magnification, and helplessness, has also been associated with worse patient-reported outcomes, independent of inflammation [6, 7]. Similarly, sleep disturbances—reported in up to 20% of patients—are strongly related to increased fatigue, impaired disease control, and decreased quality of life [8].

Despite the recognition of these mechanisms, few studies have systematically evaluated the combined impact of CS, PC, and sleep disturbance on disease burden in axSpA patients. This study aimed to investigate their prevalence and interrelationships and to assess their associations with clinical and epidemiological characteristics in comparison with those of healthy controls.

## Methods

This cross-sectional observational study included patients with axial spondyloarthritis (axSpA) and healthy controls recruited from the Rheumatology Outpatient Clinic of Hospital das Clínicas, Federal University of Pernambuco, between April 2024 and April 2025. The study protocol was approved by the local institutional ethics committee (CAAE 77066224.8.0000.8807; Approval Number 6.639.661). Oral and written informed consent was obtained from all participants in accordance with the Declaration of Helsinki.

Eligible patients were aged 18 years or older, fulfilled the 2009 classification criteria for axSpA proposed by the Assessment of SpondyloArthritis International Society (ASAS), and had been under regular follow-up at the outpatient clinic for at least three months. The exclusion criteria included active infection, malignant disease, pregnancy, chronic alcohol addiction, and cognitive impairment that interfered with comprehension or adequate completion of assessment tools.

The control group consisted of individuals aged 18 years or older who visited the Rheumatology Outpatient Clinic for any reason but had no diagnosis of rheumatic diseases, infections, malignancies, neurological conditions, psychiatric disorders, or pregnancy.

Data were collected from all participants via REDCap [9] and included demographic variables (age, sex, body mass index [BMI], smoking status), the Central Sensitization Inventory (CSI; scores 0–100, with **≥ 40** indicating clinically relevant central sensitization) [10], the Pain Catastrophizing Scale (PCS; scores 0–52, with **≥ 30** indicating clinically significant catastrophizing) [11], the 2016 American College of Rheumatology (ACR) criteria for fibromyalgia (defined by WPI ≥ 7 with SSS ≥ 5, or WPI 4–6 with SSS ≥ 9, plus widespread pain and ≥ 3 months of symptoms) [12], the Jenkins Sleep Scale (JSS; scores 0–20, higher values indicating poorer sleep quality) [13], and the Polysymptomatic Distress Scale (WPI + SSS) to quantify fibromyalgianess [14].

For the axSpA patients, additional clinical data were collected, including disease duration; HLA-B27 status; current use of nonsteroidal anti-inflammatory drugs (NSAIDs) and disease-modifying antirheumatic drugs (DMARDs); number of DMARD switches due to therapeutic failure; history of extraskeletal manifestations (inflammatory bowel disease, uveitis, or psoriasis); use of centrally acting analgesic modulators (pregabalin, gabapentin, tricyclic antidepressants, and duloxetine); and the presence of comorbidities, including systemic arterial hypertension, type 2 diabetes mellitus, depression, anxiety disorders, and fibromyalgia.

Disease-specific outcomes included the patient global assessment (PtGA), the Ankylosing Spondylitis Quality of Life Questionnaire (ASQoL; scores 0–18, with higher values indicating poorer quality of life) [15], the Bath Ankylosing Spondylitis Disease Activity Index (BASDAI; scores 0–10, with ≥ 4 indicating high disease activity), the Ankylosing Spondylitis Disease Activity Score (ASDAS; categorized as inactive < 1.3, low 1.3–2.0, high 2.1–3.5, and very high > 3.5), C-reactive protein (CRP) levels, the Bath Ankylosing Spondylitis Functional Index (BASFI; scores 0–10, with higher values reflecting greater functional limitation), the Bath Ankylosing Spondylitis Meteorology Index (BASMI; scores 0–10, with higher scores indicating more impaired spinal mobility), and the Maastricht Ankylosing Spondylitis Enthesitis Score (MASES; scores 0–13, with higher values representing more extensive enthesitis).

### Statistical analysis

The normality of continuous variables was assessed via the Shapiro–Wilk test. Variables with a normal distribution (age, CSI, and ASDAS) are presented as the means ± standard deviations (SDs) and were compared between groups (axSpA patients and controls) via the independent-samples Student’s t test. Continuous variables without a normal distribution are described as medians and interquartile ranges (IQRs, Q1–Q3) and were compared via the Mann–Whitney U test. Categorical variables are expressed as absolute and relative frequencies, with comparisons performed via Pearson’s chi-square test or Fisher’s exact test, when appropriate.

Correlation analyses between continuous variables were conducted via Spearman’s correlation coefficient, and the results are displayed in a correlation matrix heatmap. To further explore the relationships of the CSI, PCS, and JSS with disease activity, function, and quality of life, scatterplots with correlation coefficients and 95% confidence intervals were generated.

Finally, multivariable linear regression analyses were performed to identify independent predictors of the CSI, PCS, and JSS. Variables were entered into the models on the basis of their clinical relevance and results from univariate analyses. Regression coefficients (β), 95% confidence intervals, and p values are reported. Model performance was assessed by the correlation coefficient (R), adjusted R², and overall model significance.

All analyses adopted a two-sided p value < 0.05 as the threshold for statistical significance and were performed via IBM SPSS Statistics, version 29.0.2.0, and Jamovi (version 2.7.2.0). Graphical outputs, including heatmaps and scatterplots, were generated via GraphPad Prism (version 10.5.0) and Jamovi (version 2.7.2.0).

## Results

A total of 150 volunteers were evaluated, including 100 patients with axSpA and 50 control individuals. The groups were comparable in terms of age (*p* = 0.25), sex (*p* = 0.160), and smoking status (*p* = 0.665). However, patients with axSpA presented significantly higher scores for central sensitization (median CSI: 42 vs. 28; *p* < 0.001). pain catastrophizing (median PCS: 32 vs. 12.5; *p* < 0.001), and sleep disturbances (median JSS: 12 vs. 6; *p* < 0.001) compared to controls. In addition, the frequency of individuals meeting the criteria for central sensitization (59% vs. 20%; *p* < 0.001). pain catastrophizing (53% vs. 18%; *p* < 0.001). and fibromyalgia based on the 2016 ACR criteria (43% vs. 18%; *p* = 0.002) was significantly greater in the axSpA group. The full data are presented in Table 1.

**Table 1:** Comparison of demographics, clinical, and neuropsychological characteristics of patients with axial spondyloarthritis and control individuals

	axSpA (*n* = 100)		Control group (*n* = 50)			*P* value CI 95%
	*n*	%		*n*	%	
Male	75	75.0	32		64.0	0.160
Age (mean, SD)	43.6 (11.1)		41.2 (14.1)			0.25
Smoking	4	4.0	1		2.0	0.665
BMI (kg/m²) (median, IQR)	26.7 (23.9–29.8)		25.2 (23.3–26.6)			**0.019**
CSI (0 -100) (median, IQR)	42 (32–58)		28 (14–39)			**< 0.001**
PCS (0–52) (median, IQR)	32 (18.75–38.3)		12.5 (1.0–21.0)			**< 0.001**
SSJ (0–20) (median, IQR)	12.0 (5–16)		6.0 (3.0–9.0)			**< 0.001**
SC (CSI ≥ 40)	59	59.0	10		20.0	**< 0.001**
PC (PCS ≥ 30)	53	53.0	9		18.0	**< 0.001**
Fibromyalgia ACR 2016	43	43.0	9		18.0	**0.002**

BMI: body mass index; CSI: Central Sensitization Inventory; PCS: Pain Catastrophizing Scale; SSJ: Jenkins Sleep Scale; SC: central sensitization (CSI ≥ 40); PC: pain catastrophizing (PCS ≥ 30); FM ACR 2016: fibromyalgia criteria according to the 2016 ACR definition applied during the study. Data are presented as the mean (SD) or median (IQR) for continuous variables and as n (%) for categorical variables. P values refer to comparisons between groups. Comparisons between groups were performed via the Mann–Whitney U test for continuous variables and the chi-square test or Fisher’s exact test for categorical variables

The clinical characteristics of patients with axial SpA are summarized in Table 2. Among 100 patients with axSpA, 60.0% were HLA-B27 positive. The median symptom duration was 14 years (IQR 8–20), and the median disease duration was 10 years (IQR 6–18). Uveitis was reported in 24.0% of patients, and hypertension was the most common comorbidity (37.0%). A prior clinical diagnosis of fibromyalgia was documented in 6.0% of the cohort. The median BASDAI was 3.88 (IQR 2.5–6.2), and the median ASDAS was 1.74 (IQR 1.2–2.4). According to the ASDAS categories, 26.2% of the patients had low disease activity, 33.8% had moderate disease activity, 35.4% had high disease activity, and 4.6% had very high disease activity. The median BASFI, BASMI, and ASQoL scores were 5.8 (IQR 3.8–7.8), 4.0 (IQR 1.7–6.0), and 9.5 (IQR 6.0–13.0), respectively. The median FMness score was 13 (IQR 8–20.3), and the median CRP level was 0.50 mg/dL (IQR 0.2–0.7). NSAIDs were used by 28.0% of patients, and conventional DMARDs were used by 38.0%. Biologic therapy was frequent, with TNFi in 67.0% and IL-17i in 14.0% of patients.

**Table 2:** Clinical and demographic data, and treatment characteristics of patients with AxSpA

	axSpA (*n* = 100)	
	*n*	%
**HLA B27 status**		
Present	60	60.0
Absent	17	17.0
Unknown	23	23.0
**Symptom duration (years) (median**,** IQR)**	14 (8–20)	
**Disease durantion (years) (median**,** IQR)**	10 (6–18)	
**History of IBD**	4	4.0
**History of uveitis**	24	24.0
**History of psoriasis**	1	1.0
**Comorbidities**		
Hypertension	37	37.0
Type 2 diabetes	6	6.0
Fibromyalgia	6	6.0
Depression	3	3.0
Generalized anxiety disorder	3	3.0
**Active enthesitis**	13	13.0
**MASES (median**,** IQR)**	1.5 (0–4.0)	
**Active arthritis**	40	40.0
**BASDAI (median**,** IQR)**	3.88 (2.5–6.2)	
**ASDAS (median**,** IQR)**	1.74 (1.2–2.4)	
Inactive disease	17	26.2
Low disease activity	22	33.8
High disease activity	23	35.4
Very high disease activity	3	4.6
**WPI (median**,** IQR)**	7 (4–12)	
**SSS (median**,** IQR)**	6 (4–9)	
**FMness score (median**,** IQR)**	13 (8-20.3)	
**BASFI (0–10) (median**,** IQR)**	5.8 (3.8–7.8)	
**BASMI (median**,** IQR)**	4.0 (1.7-6.0)	
**ASQOL (0–18) (median**,** IQR)**	9.5 (6.0–13.0)	
**MASES (median**,** IQR)**	1.5 (0–4.0)	
**CRP mg/dL (median**,** IQR)**	0.50 (0.2–0.7)	
**Current NSAID use**	28	28.0
**Current conventional DMARD use**	38	38.0
Used alone or with NSAID	*13*	*13.0*
In combination with anti-TNF	*16*	*16.0*
In combination with anti-IL17	*9*	*9.0*
**TNFi**	67	67.0
**IL17i**	14	14.0
**JAKi i**	1	1.0

IBD = inflammatory bowel disease; MASES = Maastricht Ankylosing Spondylitis Enthesitis Score; BASDAI = Bath Ankylosing Spondylitis Disease Activity Index; ASDAS = Ankylosing Spondylitis Disease Activity Score; WPI = Widespread Pain Index; SSS = Symptom Severity Scale; FMness score = fibromyalgianess score; BASFI = Bath Ankylosing Spondylitis Functional Index; BASMI = Bath Ankylosing Spondylitis Metrology Index; ASQoL = Ankylosing Spondylitis Quality of Life; CRP = C-reactive protein; NSAIDs = nonsteroidal anti-inflammatory drugs; DMARDs = disease-modifying antirheumatic drugs; TNFi = tumor necrosis factor inhibitors; IL17i = interleukin-17 inhibitors; JAKi = Janus kinase inhibitors. The results are presented as absolute numbers and percentages or as medians (interquartile ranges) according to the variable distribution

The associations between clinical and treatment-related factors and the CSI, PCS, and JSS are presented in Table 3. CSI scores were significantly higher in females than in males (58 vs. 40; *p* = 0.004). Patients fulfilling the ACR 2016 fibromyalgia criteria presented consistently higher CSI, PCS, and JSS scores than did those without fibromyalgia (*p* < 0.001 for all). The presence of enthesitis and arthritis was also associated with higher CSI and JSS scores (all *p* < 0.05). NSAID use was linked to higher CSI and PCS scores (*p* = 0.030 and *p* = 0.024, respectively). Patients not receiving pain modulation therapy had higher CSI and JSS scores than did those receiving such treatment (*p* < 0.001 and *p* = 0.003). However, amitriptyline and gabapentinoid use was also associated with significantly higher CSI and JSS scores (all *p* ≤ 0.005). Anti-TNF therapy was related to lower JSS scores (*p* = 0.014). No significant differences were observed according to HLA-B27 status, presence of low back pain, biological switch, or duloxetine use.

**Fig. 1:**
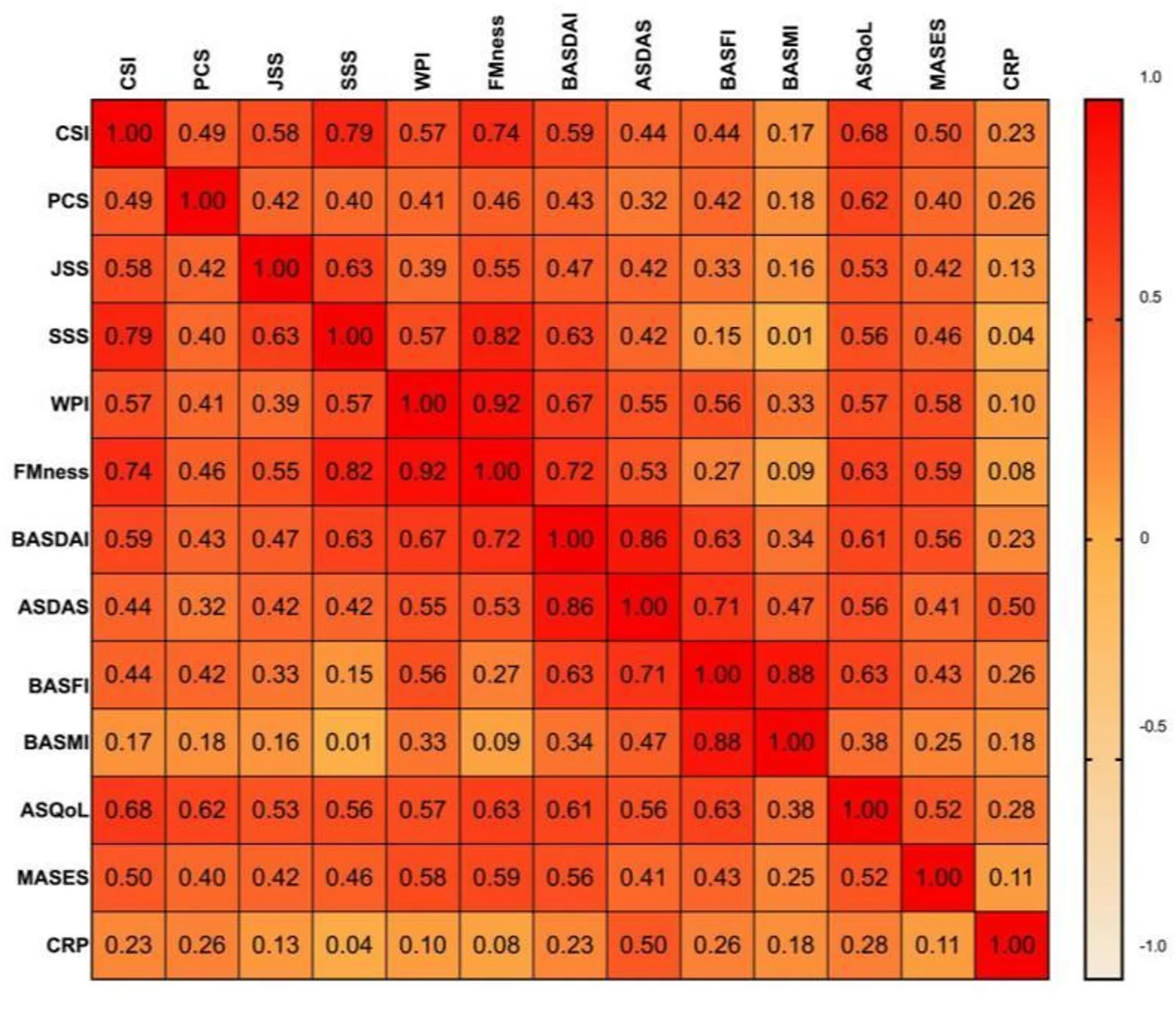
Correlation matrix of clinical, functional, and patient-reported outcomes in patients with axial spondyloarthritis. The heatmap displays pairwise correlations (Spearman’s rho) among clinical variables. Warmer colors indicate stronger positive correlations, and cooler colors indicate weaker or absent correlations. The values within each cell represent correlation coefficients. Correlations were classified as strong when rho > 0.70, moderate when between 0.40 and 0.69, and weak when < 0.40. ASDAS = Ankylosing spondylitis disease activity score, ASQoL = Ankylosing spondylitis quality of life, BASDAI = Bath ankylosing spondylitis disease activity index, BASFI = Bath ankylosing spondylitis functional index, BASMI = Bath ankylosing spondylitis metrology index, BMI = Body mass index, CRP = C-reactive protein, CSI = Central sensitization inventory, FMness = Fibromyalgia score, JSS = Jenkins sleep scale, MASES = Maastricht ankylosing spondylitis enthesitis score, PCS = Pain catastrophizing scale, SSS = Symptom severity scale, WPI = Widespread pain index

As shown in Fig. 1, the correlation matrix revealed clear patterns among the clinical and patient-reported outcomes. Disease activity measures were tightly interconnected, with very strong correlations observed between the BASDAI and ASDAS (*r* = 0.92) and between the BASFI and both the BASDAI (*r* = 0.82) and the ASDAS (*r* = 0.72). Measures of widespread pain and symptom severity also clustered closely together (WPI and SSS, *r* = 0.92). Quality of life (ASQoL) was strongly associated with both disease activity and functional limitations (*r* = 0.63 with the BASFI and BASDAI). Central sensitization (CSI) showed consistent moderate correlations with disease activity and quality of life (*r* = 0.58–0.63), whereas sleep disturbance (JSS) was moderately related to the CSI (*r* = 0.49) and WPI (*r* = 0.52). In contrast, objective markers such as CRP and BASMI displayed only weak associations with most clinical and patient-reported measures, including the BASDAI (*r* = 0.20 and *r* = 0.32, respectively). Together, these patterns outline two distinct domains within the matrix: a strongly correlated group of patient-reported outcomes (BASDAI, ASDAS, BASFI, ASQoL, CSI, WPI, SSS, JSS) and a set of objective measures (CRP, BASMI) with comparatively weaker associations.

**Table 3:** Clinical and treatment-related factors associated with the central sensitization inventary (CSI) pain catastrophizing score (PCS) and Jenkins sleep scale (JSS) score in AxSpA patients

	CSI			PCS			JSS		
	Median	IQR (25–75)	*P* value* (CI95%)	Median	IQR (25–75)	*P* value* (CI95%)	Median	IQR (25–75)	*P* value* (CI95%)
**Sex**									
Female	58	41–68	**0.004**	33	13–32	0.886	11	6–16	0.460
Male	40	31–53		32	19–41		12	4–16	
**HLA-B27**									
Positive	54	38–68	0.065	28	16–38	0.931	16	9–16	0.261
Negative	42	30–55		31	21–38		11	5–16	
**Fibromyalgia ACR 2016**									
No	40	30–54	**< 0.001**	27	14–37	**< 0.001**	9	4–16	**< 0.001**
Yes	49	36–65		34	26–39		12.5	10–16	
**Low back pain**									
No	38	26–49	0.145	24	12–41	0.561	8	5–12	0.096
Yes	44	34–58		32	19–38		12	5–16	
**Enthesitis**									
No	40	31–55	**0.009**	32	16–38	0.448	10	4–16	**0.019**
Yes	56	53–68		33	29–37		16	13–16	
**Arthritis**									
No	40	30–54	**0.025**	27	14–37	**0.028**	9	4–16	**0.022**
Yes	49	36–65		34	26–39		12.5	10–16	
**NSAID use**									
No	40	31–54	**0.030**	28	15-37.3	**0.024**	10	4–16	0.066
Yes	50	40–63		35	27-39.5		13	8–18	
**Anti-TNF use**									
No	44	36–63	0.238	33	26–41	0.051	14	10–17	**0.014**
Yes	41	31–55		28	14–37		10	4–16	
**Biological switch**									
1	42	27–64	0.322	33	25–38	0.479	9	5–16	0.308
2 or 3	63	50–65		35	32–41		16	5–16	
**Pain modulation therapy**									
No	63	54–68	**< 0.001**	33	23–37	0.671	16	16–18	**0.003**
Yes	40	31–54		31	18–39		10	5–15	
**Amitriptyline**									
No	41	32–55	**0.004**	30	17–38	0.117	11	5–16	**0.005**
Yes	69	64–69		37	33–38		19	16–19	
**Gabapentinoids**									
No	40	32–56	**< 0.001**	31	17–38	0.490	10	5–16	**< 0.001**
Yes	60	57–68		33	31–37		17	18–19	
**Duloxetin**									
No	41	31–54	0.174	32	18–38	0.319	12	5–16	0.810
Yes	58	55–67		20	26–41		11	12–18	

CSI = Central Sensitization Inventory; PCS = Pain Catastrophizing Scale; JSS = Jenkins Sleep Scale; HLA-B27 = human leukocyte antigen B27; ACR = American College of Rheumatology; NSAID = nonsteroidal anti-inflammatory drug; TNF = tumor necrosis factor. Comparisons between groups were performed via the Mann–Whitney U test, given the nonnormal distribution of the variables. The results are reported as medians with interquartile ranges (IQRs, 25th–75th percentiles)

**Fig. 2:**
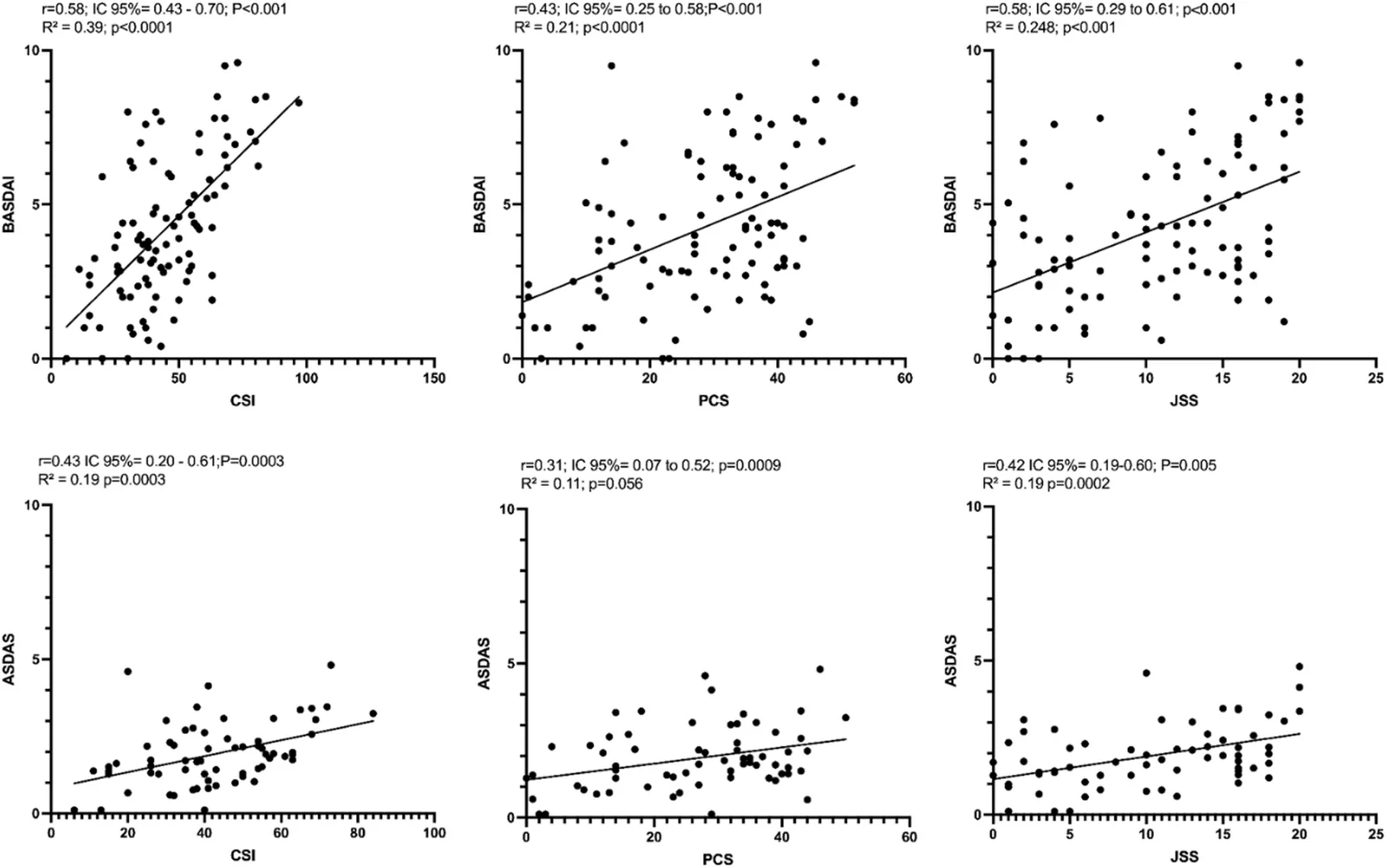
Correlations of central sensitization (CSI), pain catastrophizing (PCS), and sleep disturbance (JSS) with disease activity (BASDAI and ASDAS) in axial spondyloarthritis patients. Scatter plots with linear regression lines showing the correlations between CSI or PCS scores and the BASDAI, the Bath Ankylosing Spondylitis Disease Activity Index, and the ASDAS, the Ankylosing Spondylitis Disease Activity Score. Each plot displays the Pearson correlation coefficient (r), the 95% confidence interval (CI) for r, and the coefficient of determination (R²), derived from simple linear regression

**Fig. 3:**
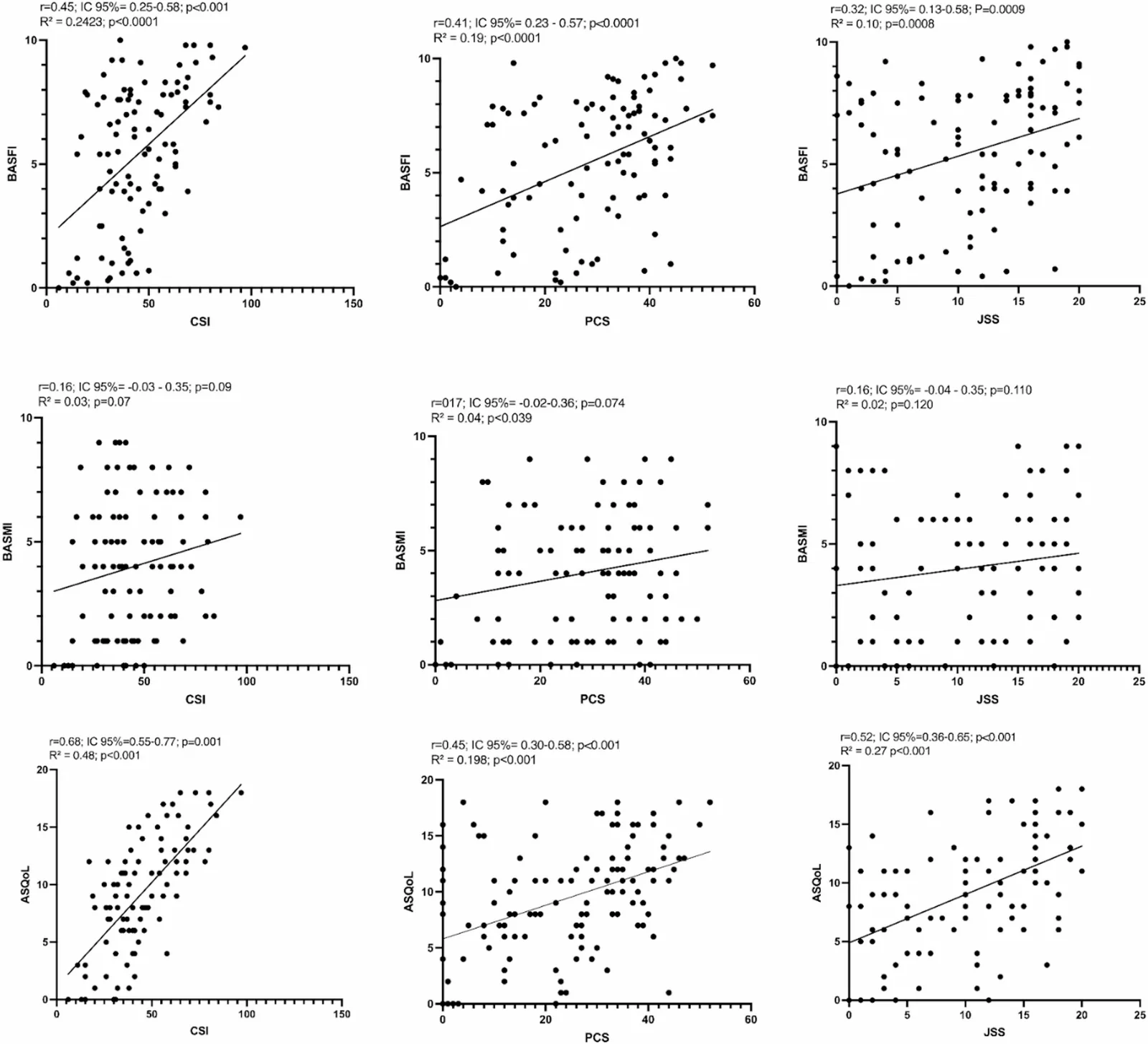
Correlations of the CSI, PCS, and JSS with functional limitations (BASFI, BASMI) and quality of life (ASQoL) in patients with axial spondyloarthritis. Scatter plots with linear regression lines showing the correlations between CSI, PCS, or JSS scores and the BASFI: Bath Ankylosing Spondylitis Functional Index and the ASQoL: Ankylosing Spondylitis Quality of Life Questionnaire. Each plot displays the Pearson correlation coefficient (r), the 95% confidence interval (CI) for r, and the coefficient of determination (R²), derived from simple linear regression

We further explored the individual contributions of the CSI, PCS, and JSS by analyzing their associations with disease activity, function, and quality of life (Figs. 2 and 3). The CSI was moderately correlated with both the BASDAI (*r* = 0.58, 95% CI 0.43–0.70; *p* < 0.001) and the ASDAS (*r* = 0.43, 95% CI 0.22–0.61; *p* = 0.0003) and was also related to functional outcomes, including the BASFI (*r* = 0.45, 95% CI 0.25–0.58; *p* < 0.001) and the BASMI (*r* = 0.45, 95% CI 0.25–0.58; *p* < 0.001). The strongest association was observed with quality of life, as measured by the ASQoL (*r* = 0.68, 95% CI 0.55–0.77; *p* < 0.001). The PCS was consistently associated with worse outcomes, correlating with the BASDAI (*r* = 0.46, 95% CI 0.25–0.58; *p* < 0.001) and ASDAS (*r* = 0.31, 95% CI 0.07–0.52; *p* = 0.0009), as well as with the BASFI (*r* = 0.41, 95% CI 0.23–0.57; *p* < 0.001), BASMI (*r* = 0.41, 95% CI 0.23–0.57; *p* < 0.001), and ASQoL (*r* = 0.45, 95% CI 0.30–0.58; *p* < 0.001). Similarly, the JSS demonstrated moderate correlations with both the BASDAI (*r* = 0.46, 95% CI 0.29–0.61; *p* < 0.001) and ASDAS (*r* = 0.42, 95% CI 0.19–0.60; *p* = 0.005) and was also associated with the BASFI (*r* = 0.32, 95% CI 0.13–0.58; *p* < 0.001), BASMI (*r* = 0.32, 95% CI 0.13–0.58; *p* < 0.001), and ASQoL (*r* = 0.52, 95% CI 0.36–0.65; *p* < 0.001).

Multivariable linear regression models evaluating the impact of pain-related variables on clinical outcomes are presented in Table 4. For the CSI model, female sex (β = 5.01, 95% CI 0.15–9.86; *p* = 0.043), SSS (β = 3.01, 95% CI 2.20–3.81; *p* < 0.001), and ASQoL (β = 1.52, 95% CI 2.03–5.90; *p* < 0.001) emerged as independent predictors, with the model explaining a large proportion of the variance (*R* = 0.844; adjusted R² = 0.703; *p* < 0.001). For PCS, ASQoL was the only significant predictor (β = 1.68, 95% CI 1.26–2.11; *p* < 0.001), with the model accounting for a moderate proportion of variance (*R* = 0.625; adjusted R² = 0.391; *p* < 0.001). The JSS model identified SSS (β = 1.06, 95% CI 0.76–1.35; *p* < 0.001) and arthritis (β = 2.09, 95% CI 0.23–3.93; *p* = 0.027) as positive predictors, whereas TNFi treatment (β = − 2.57, 95% CI − 4.48–0.65; *p* = 0.009) and pain modulation therapy (β = − 3.14, 95% CI − 5.71–0.57; *p* = 0.017) were associated with lower scores. This model also showed good explanatory capacity (*R* = 0.701; adjusted R² = 0.491; *p* < 0.001).

**Table 4:** Multivariable linear regression models evaluating the impact of pain-related variables on the clinical outcomes of patients with AxSpA

Variable		β (Coefficient)		95% CI	*P* value
**CS**					
Intercept		16.30		9.65–22.94	< 0.001
Female sex		5.01		0.15–9.86	0.043
SSS		3.01		2.20–3.81	< 0.001
ASQoL		1.52		2.03–5.90	< 0.001
*R* = 0.844; Adjusted R ^2^= 0.703; *p* < 0.001					
**PCS**					
Intercept		12.71		8.29–17.14	< 0.001
ASQoL		1.68		1.26–2.11	< 0.001
*R* = 0.625; Adjusted R ^2^= 0.391; p,0.001					
**JSS**					
Intercept		7.79		4.14–11.4	< 0.001
SSS		1.06		0.76–1.35	< 0.001
TNFi treatment		− 0.2.57		-4.48 - -0.65	0.009
Pain modulation therapy		-3.14		-5.71- -0.57	0.017
Arthritis		2.09		0.23–3.93	0.027
*R* = 0.701; Adjusted R ^2^= 0.491; *p* < 0.001					

Multivariable linear regression models were constructed for each outcome: the Central Sensitization Inventory (CSI), the Pain Catastrophizing Scale (PCS), and the Jenkins Sleep Scale (JSS). The results are presented as regression coefficients (β) with 95% confidence intervals (CIs) and p values. Model fit is expressed as the correlation coefficient (R), adjusted R², and overall model significance. ASQoL = Ankylosing Spondylitis Quality of Life; CSI = Central Sensitization Inventory; JSS = Jenkins Sleep Scale; PCS = Pain Catastrophizing Scale; SSS = Symptom Severity Scale; TNFi = Tumor Necrosis Factor inhibitor

## Discussion

In this cross-sectional study, we demonstrated that CS, PC, FM, and poor sleep quality were highly prevalent among individuals with axSpA and were consistently associated with worse disease activity, functional limitations, and impaired quality of life. These findings reinforce the concept that the burden of axSpA extends beyond objective inflammation and is strongly modulated by nociplastic and psychosocial mechanisms, which emerge as independent determinants of the overall disease impact.

Clinically relevant CS was identified in more than half of the patients, with 59% presenting CSI scores ≥ 40, aligning with prior reports of prevalence rates between 40 and 63% in similar cohorts [3, 4, 16]. PC was also markedly elevated, with median PCS scores of 32, and over half of the patients exceeded the clinically relevant threshold of 30. This prevalence surpasses that reported in other axSpA cohorts [17]; [18] and underscores the clinical significance of maladaptive cognitive-emotional processes. Notably, PC was independently correlated with higher BASDAI, ASDAS, BASFI, BASMI, and ASQoL scores, confirming its role as a key driver of pain perception, disability, and impaired treatment response [6, 7, 19, 20]. The strong interconnection between CSs and PCs observed here further highlights their reciprocal reinforcement, where nociplastic mechanisms amplify catastrophizing, which in turn perpetuates disability and worsens quality of life [21, 22].

FM was identified in 43% of patients, which is consistent with recent studies demonstrating substantial overlap between axSpA and FM, reported in at least one in six patients [23, 24]. In our cohort, FM was associated with significantly worse CSI, PCS, and JSS scores, supporting the view that FM potentiates nociplastic pain, maladaptive cognitions, and sleep disturbances, thereby amplifying disease burden in a multidimensional manner. These findings align with the contemporary framework of “fibromyalgianess,” where widespread pain and somatic symptoms converge with nociplastic processes to shape clinical outcomes [20].

In addition to categorical FM, the construct of fibromyalgianess (FMness) deserves emphasis. FMness represents a continuous spectrum of symptoms characterized by diffuse pain, somatic hypersensitivity, fatigue, and sleep disturbance, without necessarily fulfilling the full diagnostic criteria for FM. This dimensional approach has gained traction as a more sensitive reflection of nociplastic burden, capturing subthreshold or intermediate phenotypes often observed in axSpA patients. In our study, the strong clustering of WPI and SSS scores (*r* = 0.92), positioned closely with CSI and sleep disturbance measures, supports the existence of such a spectrum. These findings suggest that FM may represent a transdiagnostic phenotype overlapping with CS, mediating the link between diffuse pain, cognitive-emotional dysregulation, and impaired quality of life. Considering FMness rather than only categorical FM may provide a more nuanced understanding of disease heterogeneity and facilitate the design of individualized therapeutic strategies.

Sleep quality was consistently impaired in axSpA patients and emerged as an independent correlate of disease activity, function, and quality of life. JSS scores correlated with CSI and PCS, supporting the role of disturbed sleep as both a consequence and a mediator of central pain mechanisms [4, 25]. Importantly, TNF inhibitors were associated with better sleep scores, echoing previous studies suggesting that biologics may exert beneficial effects beyond inflammation, particularly on fatigue and sleep disturbance [26, 27].

Among the strongest associations observed, CSI correlated robustly with disease activity, function, mobility, and most prominently, quality of life. This underscores CS as a central integrator of the multidimensional disease burden in axSpA, with effects extending far beyond pain intensity [3, 16]. Consistent with prior data, CS did not correlate with disease duration, suggesting that once established, it may persist independently of symptom chronology, likely sustained by ongoing nociceptive input and diagnostic delay [28]. Higher CSI scores among women are also consistent with literature indicating a female predisposition to nociplastic pain and poorer clinical outcomes, potentially explained by neuroendocrine and psychosocial factors [29, 30].

Enthesitis and arthritis were associated with higher CSI and JSS scores, supporting the hypothesis that inflammatory pain may act as a trigger for CS and sleep disruption. Importantly, the overlap between enthesitis and FM tender points raises the possibility that some clinical enthesitis may in fact reflect nociplastic pain rather than objective inflammation [29–31], complicating clinical interpretation.

Medication patterns added further nuance: NSAID and analgesic use were more frequent among patients with higher CSI and PCS scores, likely reflecting indication bias rather than causal relationships. Similarly, patients using amitriptyline or gabapentinoids showed worse CSI and JSS scores, again consistent with greater baseline symptom burden. While pharmacological strategies remain valuable, these findings highlight their limited efficacy in targeting CS, reinforcing the need for multimodal interventions including exercise, psychological therapies, and pain neuroscience education [32].

Correlation matrix analysis revealed two distinct clusters: (i) self-reported outcomes (BASDAI, ASDAS, BASFI, ASQoL, CSI, PCS, JSS, WPI, SSS), highly interdependent, and (ii) objective markers (CRP, BASMI), only weakly correlated with the former. This dichotomy underscores the importance of integrating both perspectives—biological and patient-reported—when assessing disease burden, as objective inflammation alone cannot explain the lived experience of axSpA.

Regression models further refined these relationships. For CS, female sex, somatic symptom severity, and poorer quality of life were independent predictors, collectively explaining substantial variance. PC was uniquely predicted by ASQoL, underscoring its central cognitive-emotional role in shaping disease experience. For sleep disturbance, arthritis, somatic symptoms, and therapeutic interventions (anti-TNF and pain modulators) emerged as independent determinants. Together, these models highlight the complex interplay between nociplastic, psychosocial, and inflammatory factors in axSpA.

This study has limitations. The cross-sectional design precludes causal inference, and unmeasured factors such as physical activity, socioeconomic status, and education may have influenced outcomes. Additionally, CS prevalence in controls was somewhat higher than typically reported, possibly reflecting unmeasured psychosocial or lifestyle variables. Nonetheless, strengths include the large sample size, the use of validated instruments, the inclusion of a control group, and robust multivariate analyses.

In conclusion, our findings demonstrate that CS, PC, FM, FMness, and sleep disturbance are highly prevalent and clinically meaningful in axSpA patients, exerting independent and overlapping effects on disease activity, function, and quality of life. These results emphasize the need for a multidimensional approach to disease assessment and management, incorporating strategies that target nociplastic and psychosocial domains alongside conventional anti-inflammatory therapy. Addressing these mechanisms may be pivotal for improving long-term outcomes and optimizing patient-centered care in axSpA patients.

## Data Availability

No datasets were generated or analysed during the current study.
